# Supplementary material for: Efficacy and Safety of Non-Insulin Antidiabetic Drugs in Cats: A Systematic Review
Source: Animals (Basel). 2025 Aug 31;15(17):2561. doi: 10.3390/ani15172561 (PMC12427183; doi:10.3390/ani15172561)
Supplement: Supplementary file 1 [file animals-15-02561-s001.zip › animals-3788076-supplementary.pdf]

Supplementary Material: Detailed Risk of Bias Assessment for Included Studies

This supplementary document provides a detailed, study-by-study assessment of the risk of bias for the 20 studies included in the systematic review "Efficacy and Safety of Non-Insulin Antidiabetic Drugs in Cats". The appropriate risk of bias tool was selected for each study based on its design: the Cochrane Risk of Bias 2 (RoB 2) tool for randomized controlled trials (RCTs) and the Risk Of Bias In Non-randomised Studies of Interventions (ROBINS-I) tool for non-randomized studies.

Risk of Bias Analysis for Nelson et al. (1993)

- Tool Used: ROBINS-I (Prospective Non-Randomized Intervention Study).
- Domain 1: Bias due to confounding: Moderate. Absence of a concurrent comparison group; factors changing over time were not controlled.
- Domain 2: Bias in selection of participants: Low Risk. Clearly defined inclusion and exclusion criteria, with no indication of inappropriate selection.
- Domain 3: Bias in classification of interventions: Low Risk. Intervention (Glipizide 5 mg, PO, q12h) clearly defined.
- Domain 4: Bias due to deviations from intended interventions: Moderate. Some non-responding cats were switched to insulin, and varied diets (high and low fiber) were used.
- Domain 5: Bias due to missing data: Low Risk. Minimal missing data (2 of 20 cats) without clear description of handling, but likely small impact.
- Domain 6: Bias in measurement of outcomes: Moderate. Unblinded study; final response classification partly dependent on subjective owner satisfaction.
- Domain 7: Bias in selection of reported result: Low Risk. Transparent reporting of all response groups and adverse effects, with no apparent attempt to hide negative results.
- **OVERALL RISK OF BIAS: Moderate.**

Risk of Bias Analysis for Feldman et al. (1997)

- Tool Used: ROBINS-I (Prospective Non-Randomized Study).
- Domain 1: Bias due to confounding: Moderate. Absence of a parallel control group; factors changing over time were not controlled.
- Domain 2: Bias in selection of participants: Low Risk. Clear and well-defined inclusion criteria, systematic selection.
- Domain 3: Bias in classification of interventions: Low Risk. Intervention (Glipizide) clearly defined.
- Domain 4: Bias due to deviations from intended interventions: Serious. 56% of cats were "disqualified" and switched to insulin due to treatment failure, introducing significant bias.
- Domain 5: Bias due to missing data: Low Risk. All cats accounted for, no unexplained missing data.
- Domain 6: Bias in measurement of outcomes: Moderate. Final response classification was "arbitrary" and depended on owner satisfaction in an unblinded study.
- Domain 7: Bias in selection of reported result: Low Risk. Transparent reporting of a full range of results, including failures and adverse effects.
- **OVERALL RISK OF BIAS: Serious.**

Risk of Bias Analysis for Cohn et al. (1999)

- Tool Used: ROBINS-I (Non-Randomized Placebo-Controlled Clinical Trial).
- Domain 1: Bias due to confounding: Low Risk. Use of a concurrent placebo control group, and non-obese groups were well-matched initially.
- Domain 2: Bias in selection of participants: Low Risk. Healthy cats with clear selection criteria.
- Domain 3: Bias in classification of interventions: Low Risk. Intervention (Chromium picolinate) and placebo clearly specified.
- Domain 4: Bias due to deviations from intended interventions: Low Risk. No reported withdrawals or deviations from the protocol.
- Domain 5: Bias due to missing data: Low Risk. No missing data mentioned.
- Domain 6: Bias in measurement of outcomes: Moderate. Blinding was not mentioned, but outcomes were objective laboratory measurements (glucose, insulin).
- Domain 7: Bias in selection of reported result: Low Risk. Transparent reporting of a negative result (lack of effect), indicating absence of selective reporting bias.
- **OVERALL RISK OF BIAS: Moderate.**

Risk of Bias Analysis for Hoenig et al. (2000)

- Tool Used: RoB 2 (Randomized Controlled Trial, experimental model).
- Domain 1: Bias in the randomization process: Some Concerns. Randomization mentioned, but no specific method described or sequence concealment.
- Domain 2: Bias due to deviations from intended interventions (Blinding): Low Risk. Likely open-label, but primary outcomes are objective (histopathology), minimizing impact.
- Domain 3: Bias due to missing outcome data: Low Risk. All 8 cats completed the 18-month study.
- Domain 4: Bias in measurement of the outcome: Some Concerns. Not specified if the pathologist evaluating amyloid was blinded, which could introduce bias.
- Domain 5: Bias in selection of the reported result: Low Risk. Results focused on the study's primary objective and reported comprehensively for both groups.
- **OVERALL RISK OF BIAS: Some Concerns.**

Risk of Bias Analysis for Hoenig & Ferguson (2003)

- Tool Used: RoB 2 (Randomized Placebo-Controlled Trial).
- Domain 1: Bias in the randomization process: Some Concerns. Randomization mentioned with blocking by weight, but no specific method or concealment described.
- Domain 2: Bias due to deviations from intended interventions (Blinding): Low Risk. Used identical placebo capsules; no major protocol deviations reported.
- Domain 3: Bias due to missing outcome data: Low Risk. Minor missing data (1 of 9 cats) due to technical issue unrelated to treatment.
- Domain 4: Bias in measurement of the outcome: Low Risk. Objective laboratory measurements (glucose, insulin curves, lipids); low risk of bias even without explicit blinding of technical staff.
- Domain 5: Bias in selection of the reported result: Low Risk. Comprehensive reporting of pre-specified results.
- **OVERALL RISK OF BIAS: Some Concerns.**

Risk of Bias Analysis for Mazzaferro et al. (2003)

- Tool Used: ROBINS-I (Non-Randomized Controlled Clinical Trial).
- Domain 1: Bias due to confounding: Serious. Non-random allocation and highly imbalanced groups (18 vs. 6), high risk of systematic differences not controlled.
- Domain 2: Bias in selection of participants: Low Risk. Clear inclusion and exclusion criteria for diabetic cats.
- Domain 3: Bias in classification of interventions: Low Risk. Intervention (Acarbose + low-carb diet) and control clearly defined.
- Domain 4: Bias due to deviations from intended interventions: Moderate. All cats received concurrent insulin therapy with monthly dose adjustments, confounding the effect of acarbose.
- Domain 5: Bias due to missing data: Low Risk. No missing data mentioned.
- Domain 6: Bias in measurement of outcomes: Moderate. Unblinded study; "responder" classification based on unblinded veterinarian's decision.
- Domain 7: Bias in selection of reported result: Low Risk. Transparent reporting of responders and non-responders, with conclusion of minimal acarbose effect, indicating no positive reporting bias.
- **OVERALL RISK OF BIAS: Serious.**

Risk of Bias Analysis for Nelson et al. (2004)

- Tool Used: ROBINS-I (Prospective Uncontrolled Clinical Trial / Case Series).
- Domain 1: Bias due to confounding: Serious. Absence of a control group; results compared before vs. after treatment, which doesn't control for time-dependent factors.
- Domain 2: Bias in selection of participants: Low Risk. Clear diagnostic criteria, but extremely small sample size (n=5) severely limits generalizability.
- Domain 3: Bias in classification of interventions: Low Risk. Intervention (Metformin) and dose escalation protocol clearly described.
- Domain 4: Bias due to deviations from intended interventions: Moderate. Protocol allowed switching to insulin for non-responders; one cat died during the study.
- Domain 5: Bias due to missing data: Low Risk. All 5 cats accounted for in results.
- Domain 6: Bias in measurement of outcomes: Moderate. Open-label study (unblinded); success evaluation combined objective results with subjective owner opinion.
- Domain 7: Bias in selection of reported result: Low Risk. Transparent reporting of successes, failures, and death, with no apparent negative reporting bias.
- **OVERALL RISK OF BIAS: Serious.**

Risk of Bias Analysis for Clark et al. (2014)

- Tool Used: RoB 2 (Randomized Placebo-Controlled 3-way Crossover Trial).
- Domain 1: Bias in the randomization process: Some Concerns. Randomization mentioned, but no specific method of sequence generation or concealment described.
- Domain 2: Bias due to deviations from intended interventions (Blinding): Low Risk. Crossover design with adequate washout period and placebo use.
- Domain 3: Bias due to missing outcome data: Low Risk. Minimal missing data (1 cat) due to cause unrelated to treatment.
- Domain 4: Bias in measurement of the outcome: Low Risk. Objective laboratory measurements (insulin sensitivity, lipids, hormones); low risk of bias even without explicit blinding.
- Domain 5: Bias in selection of the reported result: Low Risk. Transparent and comprehensive reporting of effects for both doses compared to placebo.
- **OVERALL RISK OF BIAS: Some Concerns.**

Risk of Bias Analysis for Riederer et al. (2016) and Krämer et al. (2020)

- Tool Used: ROBINS-I (Non-Randomized Placebo-Controlled Clinical Trial with alternate assignment).
- Domain 1: Bias due to confounding: Serious. Alternate (non-random) assignment, and sequence known to veterinarian, preventing initial group comparability.
- Domain 2: Bias in selection of participants: Low Risk. Well-defined inclusion and exclusion criteria for newly diagnosed diabetic cats.
- Domain 3: Bias in classification of interventions: Low Risk. Interventions (Exenatide ER or saline placebo injection) clearly defined.
- Domain 4: Bias due to deviations from intended interventions (Blinding): Moderate. Owners were blinded, but supervising veterinarians were not (they adjusted insulin dose), potentially introducing bias in management.
- Domain 5: Bias due to missing data: Low Risk. All 30 cats accounted for, no missing data reported.
- Domain 6: Bias in measurement of outcomes: Moderate. Primary outcomes (remission, metabolic control) were objective, but the final decision on insulin adjustment relied on the unblinded veterinarian.
- Domain 7: Bias in selection of reported result: Low Risk. Comprehensive reporting of all primary outcomes, including non-significant findings, and open discussion of limitations.
- **OVERALL RISK OF BIAS: Serious.**

Risk of Bias Analysis for Scuderi et al. (2018)

- Tool Used: RoB 2 (Randomized, Double-Blinded, Placebo-Controlled, Crossover Trial).
- Domain 1: Bias in the randomization process: Low Risk. Used a "random number generator" for allocation, a robust method.
- Domain 2: Bias due to deviations from intended interventions (Blinding): Low Risk. "Double-blinded" study, with investigators and owners blinded and identical placebo.
- Domain 3: Bias due to missing outcome data: Low Risk. All 8 cats completed the study, no losses or withdrawals reported.
- Domain 4: Bias in measurement of the outcome: Low Risk. Evaluators were blinded, and measured outcomes (insulin dose, weight, hormones) were objective or protected by blinding.
- Domain 5: Bias in selection of the reported result: Low Risk. Transparent reporting of both statistically significant and non-significant results.
- **OVERALL RISK OF BIAS: Low Risk.**

Risk of Bias Analysis for Hoenig et al. (2018)

- Tool Used: ROBINS-I (Non-Randomized Placebo-Controlled Clinical Trial).
- Domain 1: Bias due to confounding: Low Risk. Used a concurrent placebo control group, and groups were stratified by age, sex, and weight, showing good baseline comparability.
- Domain 2: Bias in selection of participants: Low Risk. Healthy obese cats from a research colony with clear selection criteria.
- Domain 3: Bias in classification of interventions: Low Risk. Intervention (Velagliflozin) and placebo clearly defined.
- Domain 4: Bias due to deviations from intended interventions: Low Risk. No reported withdrawals or deviations from the protocol.
- Domain 5: Bias due to missing data: Low Risk. No missing data mentioned; data for all 12 cats appeared to be analyzed.
- Domain 6: Bias in measurement of outcomes: Moderate. Blinding was not mentioned, but all reported outcomes were objective laboratory measurements.
- Domain 7: Bias in selection of reported result: Low Risk. Comprehensive and balanced reporting of both significant and non-significant results.
- **OVERALL RISK OF BIAS: Moderate.**

Risk of Bias Analysis for Leal et al. (2022)

- Tool Used: RoB 2 (Randomized Clinical Trial).
- Domain 1: Bias in the randomization process: Some Concerns. Randomization by "simple drawing," which may not guarantee concealment of allocation sequence.
- Domain 2: Bias due to deviations from intended interventions (Blinding): High Risk. Study was unblinded for owners and veterinarians, a significant source of performance bias.
- Domain 3: Bias due to missing outcome data: Low Risk. All 28 cats accounted for, no withdrawals mentioned.
- Domain 4: Bias in measurement of the outcome: Low Risk. Laboratory analyzing insulin samples was blinded, reducing measurement bias for this outcome.
- Domain 5: Bias in selection of the reported result: Low Risk. Transparent reporting of a negative primary outcome, indicating no selective reporting bias.
- **OVERALL RISK OF BIAS: High Risk.**

Risk of Bias Analysis for Benedict et al. (2022)

- Tool Used: ROBINS-I (Prospective Uncontrolled Clinical Trial / Case Series).
- Domain 1: Bias due to confounding: Serious. Absence of a control group, inability to control for time-dependent factors or Hawthorne effect.
- Domain 2: Bias in selection of participants: Low Risk. Clear inclusion/exclusion criteria, but extremely small sample size (n=5) severely limits generalizability.
- Domain 3: Bias in classification of interventions: Low Risk. Intervention (Bexagliflozin) clearly described, despite some dose variation.
- Domain 4: Bias due to deviations from intended interventions: Moderate. Protocol explicitly required insulin reduction at study start, confounding the drug's effect.
- Domain 5: Bias due to missing data: Low Risk. All cats completed the study.
- Domain 6: Bias in measurement of outcomes: Moderate. Open-label study (unblinded), potentially influencing insulin adjustment decisions, though outcomes were objective.
- Domain 7: Bias in selection of reported result: Low Risk. Balanced reporting of significant and non-significant results.
- **OVERALL RISK OF BIAS: Serious.**

Risk of Bias Analysis for Hadd et al. (2023)

- Tool Used: ROBINS-I (Prospective, Open-Label, Historically Controlled Clinical Trial).
- Domain 1: Bias due to confounding: Serious. Absence of a concurrent comparison group, making it impossible to solely attribute improvement to the drug.
- Domain 2: Bias in selection of participants: Low Risk. Large cohort (84 cats) from multiple sites with detailed inclusion/exclusion criteria.
- Domain 3: Bias in classification of interventions: Low Risk. Intervention (Bexagliflozin) clearly defined.
- Domain 4: Bias due to deviations from intended interventions: Moderate. Open-label study; recommended but non-obligatory low-carb diet introduced variability in co-interventions.
- Domain 5: Bias due to missing data: Low Risk. All cats accounted for; evaluable cats explained and withdrawn cats handled as therapeutic failures.
- Domain 6: Bias in measurement of outcomes: Moderate. Open-label study; primary efficacy outcome included subjective owner evaluation, which can be biased.
- Domain 7: Bias in selection of reported result: Low Risk. Comprehensive and transparent reporting of successes, failures, and extensive details on serious adverse events (DKA).
- **OVERALL RISK OF BIAS: Serious.**

Risk of Bias Analysis for Niessen et al. (2024)

- Tool Used: RoB 2 (Randomized, Open-Label, Active-Controlled, Non-Inferiority Trial).
- Domain 1: Bias in the randomization process: Low Risk. Used "permuted-block randomization lists," a robust method.
- Domain 2: Bias due to deviations from intended interventions (Blinding): Some Concerns. "Open label" design (unblinded), which may introduce performance bias.
- Domain 3: Bias due to missing outcome data: Some Concerns. Missing data handled with "last-observation-carried-forward" (LOCF), a method that can potentially bias results.
- Domain 4: Bias in measurement of the outcome: Some Concerns. "Open label" study with subjective clinical outcome evaluations by unblinded individuals.
- Domain 5: Bias in selection of the reported result: Low Risk. Clearly defined primary outcome reported comprehensively and transparently, including adverse events.
- **OVERALL RISK OF BIAS: Some Concerns.**

Risk of Bias Analysis for Behrend et al. (2024)

- Tool Used: ROBINS-I (Prospective, Open-Label, Baseline-Controlled Clinical Trial).
- Domain 1: Bias due to confounding: Serious. Absence of a concurrent comparison group, making causal attribution impossible.
- Domain 2: Bias in selection of participants: Low Risk. Very large cohort (252 cats) from multiple clinics with detailed criteria.
- Domain 3: Bias in classification of interventions: Low Risk. Intervention (Velagliflozin) clearly defined.
- Domain 4: Bias due to deviations from intended interventions: Moderate. Open-label study; no standardized specific diet.
- Domain 5: Bias due to missing data: Low Risk. All cats accounted for, no unexplained missing data.
- Domain 6: Bias in measurement of outcomes: Moderate. Open-label study with subjective owner evaluation of clinical signs.
- Domain 7: Bias in selection of the reported result: Low Risk. Comprehensive and transparent reporting of efficacy and adverse events, including DKA.
- **OVERALL RISK OF BIAS: Serious.**

Risk of Bias Analysis for Clark et al. (2012)

- Tool Used: RoB 2 (Randomized Crossover Trial, pharmacokinetic study).
- Domain 1: Bias in the randomization process: Some Concerns. Randomization mentioned, but no specific method of sequence generation described.
- Domain 2: Bias due to deviations from intended interventions (Blinding): Low Risk. Pharmacokinetic study with highly objective outcomes; blinding is less relevant.
- Domain 3: Bias due to missing outcome data: Low Risk. All 12 cats accounted for, no losses mentioned.
- Domain 4: Bias in measurement of the outcome: Low Risk. Objective laboratory measurements; low risk of bias.
- Domain 5: Bias in selection of the reported result: Low Risk. Comprehensive reporting of pharmacokinetic parameters.
- **OVERALL RISK OF BIAS: Some Concerns.**

Risk of Bias Analysis for Hall et al. (2015)

- Tool Used: RoB 2 (Randomized, Placebo-Controlled, Crossover Trial).
- Domain 1: Bias in the randomization process: Some Concerns. Randomization mentioned, but no specific method of sequence generation or concealment described.
- Domain 2: Bias due to deviations from intended interventions (Blinding): Low Risk. Placebo-controlled and crossover design; objective outcomes minimize the risk of influence from lack of explicit blinding.
- Domain 3: Bias due to missing outcome data: Low Risk. All 8 cats accounted for, no missing data reported.
- Domain 4: Bias in measurement of the outcome: Low Risk. Objective laboratory measurements.
- Domain 5: Bias in selection of the reported result: Low Risk. Comprehensive and balanced reporting of pharmacokinetic and pharmacodynamic parameters.
- **OVERALL RISK OF BIAS: Some Concerns.**

Risk of Bias Analysis for Hoelmkjaer et al. (2016)

- Tool Used: RoB 2 (Randomized, Placebo-Controlled, Double-Blinded, Crossover Trial).
- Domain 1: Bias in the randomization process: Some Concerns. Randomization mentioned, but no specific method of sequence generation described.
- Domain 2: Bias due to deviations from intended interventions (Blinding): Low Risk. Explicitly "blinded" for cats, owners, and investigators; used placebo.
- Domain 3: Bias due to missing outcome data: Low Risk. Withdrawal of 1 cat justified and unrelated to study; minimal missing data.
- Domain 4: Bias in measurement of the outcome: Low Risk. Evaluators were blinded, and outcomes were objective.
- Domain 5: Bias in selection of the reported result: Low Risk. Transparent reporting of both significant and non-significant effects.
- **OVERALL RISK OF BIAS: Some Concerns.**

Risk of Bias Analysis for Rudinsky et al. (2015)

- Tool Used: RoB 2 (Randomized Crossover Design).
- Domain 1: Bias in the randomization process: Some Concerns. Randomization mentioned, but no specific method of sequence generation or concealment described.
- Domain 2: Bias due to deviations from intended interventions (Blinding): Low Risk. Placebo-controlled study; objective pharmacokinetic/pharmacodynamic outcomes are less susceptible to bias from explicit blinding.
- Domain 3: Bias due to missing outcome data: Low Risk. All 6 cats that started the study were accounted for.
- Domain 4: Bias in measurement of the outcome: Low Risk. Objective laboratory measurements.
- Domain 5: Bias in selection of the reported result: Low Risk. Comprehensive and balanced reporting of pharmacokinetic and pharmacodynamic parameters.
- **OVERALL RISK OF BIAS: Some Concerns.**
